# The Effects of Bilirubin and Lumirubin on the Differentiation of Human Pluripotent Cell-Derived Neural Stem Cells

**DOI:** 10.3390/antiox10101532

**Published:** 2021-09-27

**Authors:** Nikola Capková, Veronika Pospíšilová, Veronika Fedorová, Jan Raška, Kateřina Pospíšilová, Matteo Dal Ben, Aleš Dvořák, Jitka Viktorová, Dáša Bohačiaková, Libor Vítek

**Affiliations:** 1 Institute of Medical Biochemistry and Laboratory Diagnostics, Faculty General Hospital and 1st Faculty of Medicine, Charles University, 110 00 Prague, Czech Republic; nikola.capkova@lf1.cuni.cz (N.C.); katerina.pospisilova@lf1.cuni.cz (K.P.); dalben.matteo@yahoo.it (M.D.B.); ales.dvorak@lf1.cuni.cz (A.D.); 2 Department of Histology and Embryology, Faculty of Medicine, Masaryk University, 601 77 Brno, Czech Republic; veronika.pospisilova@med.muni.cz (V.P.); 436702@mail.muni.cz (V.F.); raska@med.muni.cz (J.R.); bohaciakova@med.muni.cz (D.B.); 3 Department of Biochemistry and Microbiology, University of Chemistry and Technology, 166 28 Prague, Czech Republic; Jitka.Prokesova@vscht.cz; 4 International Clinical Research Center (ICRC), St. Anne’s University Hospital, 656 91 Brno, Czech Republic; 5 4th Department of Internal Medicine, Faculty General Hospital and 1st Faculty of Medicine, Charles University, 110 00 Prague, Czech Republic

**Keywords:** bilirubin, neurodevelopment, phototherapy

## Abstract

The ‘gold standard’ treatment of severe neonatal jaundice is phototherapy with blue–green light, which produces more polar photo-oxidation products that are easily excreted via the bile or urine. The aim of this study was to compare the effects of bilirubin (BR) and its major photo-oxidation product lumirubin (LR) on the proliferation, differentiation, morphology, and specific gene and protein expressions of self-renewing human pluripotent stem cell-derived neural stem cells (NSC). Neither BR nor LR in biologically relevant concentrations (12.5 and 25 µmol/L) affected cell proliferation or the cell cycle phases of NSC. Although none of these pigments affected terminal differentiation to neurons and astrocytes, when compared to LR, BR exerted a dose-dependent cytotoxicity on self-renewing NSC. In contrast, LR had a substantial effect on the morphology of the NSC, inducing them to form highly polar rosette-like structures associated with the redistribution of specific cellular proteins (β-catenin/N-cadherin) responsible for membrane polarity. This observation was accompanied by lower expressions of NSC-specific proteins (such as SOX1, NR2F2, or PAX6) together with the upregulation of phospho-ERK. Collectively, the data indicated that both BR and LR affect early human neurodevelopment in vitro, which may have clinical relevance in phototherapy-treated hyperbilirubinemic neonates.

## 1. Introduction

Bilirubin (BR) is the end product of the heme degradation pathway. While in the past BR was considered a potentially neurotoxic waste product of heme catabolism, numerous in vitro, in vivo, and clinical studies over recent decades have demonstrated that when it is only mildly elevated it is also an endogenous antioxidant with potent anti-inflammatory and cytoprotective effects, mediated via true endocrine mechanisms [1].

Elevated serum BR concentrations are present in most newborn infants [2]. The physiological neonatal jaundice spontaneously resolves within a few days after birth, and it is believed to even have a protective role against pathological conditions associated with increased oxidative stress [3,4]. However, severely jaundiced neonates (i.e., with hyperbilirubinemia above 340 μmol/L) are in danger of serious health risks, which may lead to bilirubin encephalopathy or even death [5]. BR is capable of binding to myelin-rich membranes, making neurons the principal target cells, with multiple biological consequences including motor and intellectual disorders, chronic BR encephalopathy, hearing deficits, or paralysis of the oculomotor muscles [6].

Phototherapy (PT) with blue–green light (400–520 nm) is the worldwide gold standard for the treatment of severe neonatal jaundice. During this therapeutic approach, BR is transformed into more polar and easily excreted BR photoproducts [7], with *Z*-lumirubin (LR), *E,Z*-BR, and *Z,E*-BR being the principal compounds. Additional BR photoproducts include monopyrrolic (*Z*-BOX A-D), dipyrrolic (propentdyopents), and tripyrrolic (biopyrrin A/B) compounds [8,9]. Although PT is generally believed to be a safe therapeutic procedure, intensive PT of extremely low birth-weight (ELBW) newborn infants has recently been reported to be associated with an increased risk of allergies, diabetes, and even cancer and overall mortality (for reviews see [10,11]). These surprising recent observations might be due to the possible negative effects of BR photoproducts on the developing newborn brain. In fact, Kranc et al. showed in their in vivo and in vitro experimental models that monopyrrolic BOXes can have vasoconstrictive effects [12]. In one of our own studies, we demonstrated the striking upregulation of proinflammatory cytokines in organotypic rat hippocampal slices after short-term exposure to LR [13]. Nevertheless, in another of our recent in vitro studies, LR exerted beneficiary effects on metabolic and oxidative stress markers when compared to BR [14]. Based on these observations, BR photoproducts could possibly affect early human neurodevelopment, although no scientific data relevant to this assumption are currently available. 

Hence, the aim of this study was to compare the effects of BR and its major photo-oxidation product (LR) on the proliferation, differentiation, morphology, and specific gene and protein expressions of self-renewing neural stem cells (NSC) derived from human pluripotent stem cells (hPSC) [15,16,17,18]. These NSC lines have the ability to self-renew and terminally differentiate into neurons and glia [15], and thus they represent a biologically and developmentally relevant surrogate human model to study the influence of the potentially biologically active compounds on these processes.

## 2. Materials and Methods

All chemicals and reagents were obtained from Sigma-Aldrich (St. Louis, MO, USA), unless otherwise specified.

### 2.1. Unconjugated BR and LR Preparation

Unconjugated BR was purified according to McDonagh and Assisi 1972 [19], and LR was prepared as previously described [20] with slight modifications. Briefly, unconjugated BR was dissolved in 2 mL of NaOH (0.1 mol/L), immediately neutralized with 1 mL of H_3_PO_4_ (0.1 mol/L) and gently mixed with 7 mL of human serum albumin in PBS (phosphate buffer saline) (660 µmol/L). The final concentration of unconjugated BR was 480 µmol/L. The mixture was irradiated for 120 min at 460 nm using a Lilly phototherapeutic device (TSE Medical, Czech Republic) at 70 μW/(cm^2^·nm). The irradiated solution was mixed with ammonium acetate in methanol (0.1 mol/L; 1:1 *v*/*v*) and then vortexed. Following the Folch extraction, the solution was mixed with chloroform and vortexed intensively for at least 30 s, followed by the addition of water; the mixture was then briefly vortexed and centrifuged at 3000× *g* for 10 min at 4 °C. After the separation, the lower chloroform phase containing LR was transferred to the glass flask, and chloroform was evaporated by a vacuum rotary evaporator (RVO 200 A, INGOS, Czech Republic). The residue was dissolved in chloroform/methanol solution and separated by thin-layer chromatography (TLC) on silica gel plates (TLC silica gel 60 plates, 0.5 × 200 × 200 mm, Merck, Darmstadt, Germany); mobile phase = chloroform/methanol/water (40:9:1, *v/v/v*). The LR was isolated from scraped silica gel by methanol, dried under a stream of nitrogen, and its purity and identity verified by both HPLC and LC-MS/MS analyses (19). For all experiments, the BR was dissolved in DMSO (dimethyl sulphoxide) (final concentration of DMSO = 0.1%), whereas the LR was dissolved in PBS.

### 2.2. Cell Cultivation and Differentiation to Neurons

An established NSC cell line (CoMo-NSC) was derived from human embryonic stem cells (cell line ESI-017, ESI BIO, Alameda, CA, USA) as described previously [15]. These self-renewing NSC were cultured at 37 °C in a 5% CO_2_ atmosphere, on the cultivation plates coated with poly-L-ornithine and laminin (Thermo Fisher Scientific, Waltham, MA, USA) in the basic NSC (standard growth) medium consisting of DMEM/F12 (Dulbecco’s Modified Eagle Medium), 1% Glutamax, 1% non-essential amino acids, 0.5% N_2_ supplement, 0.5% B27 supplement without vitamin A, FGF2 (fibroblast growth factor 2) recombinant human protein (Thermo Fisher Scientific, Waltham, MA, USA), and 5 µL/mL of Zell shield cell culture contamination preventive solution (Minerva Biolabs, Berlin, Germany). The first step of the coating was the addition of 20 µg/mL poly-L-ornithine diluted in PBS. After 15 min of incubation at room temperature, the plates were washed three times with PBS, coated with 4.2 µg/mL laminin diluted in PBS, and incubated for 30 min at room temperature. For passage, the cells were detached from the surface by Accutase (Thermo Fisher Scientific, Waltham, MA, USA) and harvested with the basic NSC medium without FGF2. After centrifugation at 200× *g* for 5 min, the cells were resuspended in NSC medium, counted in a hemocytometer, and immediately seeded in the appropriate density on plates coated with poly-l-ornithine and laminin for the experiments.

For terminal differentiation studies, the NSC on day 0 were seeded at the density of 25,000/cm^2^ on a 24-well plate with cover glasses, and cultured at 37 °C in a 5% CO_2_ atmosphere. From day 3, the cells were treated for 4 days with BR (12.5 µM) and LR (12.5 and 25 µM), PBS (1%), and DMSO (0.1%) in basic NSC medium without FGF2, with the addition of brain-derived neurotrophic factor (BDNF) and glial cell line-derived neurotrophic factor (GDNF) (20 ng/mL) (Peprotech, London, UK), cAMP (300 ng/mL) and Zell shield solution (5 µL/mL). The medium was changed every day. On day 7, the cells were cultured in basic NSC medium without FGF2 and with Zell shield solution (5 µL/mL) and DAPT γ-secretase inhibitor (2.5 μM; Selleckchem, Houston, TX, USA). NSC were cultured in this medium for the next 10 days and the medium was changed every second day. The experiment lasted for a total of 17 days.

### 2.3. Additional In Vitro Studies

Unless otherwise specified, in all of the other in vitro studies the NSC were seeded at the density of 12,000/cm^2^ in five 96-well plates. One day after seeding, the NSC were treated for 96 h with fresh basic medium and the addition of BR (6.25, 12.5, 25, and 50 µM), LR (6.25, 12.5, 25, and 50 µM), with PBS (1%)/DMSO (0.1%) as the negative control. The medium was changed every day.

#### 2.3.1. Growth Curve Analysis

Each day one of the 96-well plates was fixed with 4% paraformaldehyde, then washed two times with PBS, stained with 0.1% crystal violet for 60 min at room temperature, and washed three times with deionized water. Subsequently, 33% acetic acid was added for 20 min at room temperature while being shaken. Absorbance was measured at 570 nm with a Synergy HTX spectrophotometer (BioTek Instruments, Inc., Winooski, VT, USA).

#### 2.3.2. Cytotoxicity Assay

A 96-well plate was fixed with 4% paraformaldehyde and washed three times with PBS. Nuclei were labeled with Hoechst 33342 solution (Thermo Fisher Scientific, Waltham, MA, USA), diluted to a final concentration of 5 µg/mL in PBS, incubated for 20 min, and washed three times with PBS.

Cell imaging was performed on an ImageXpress Micro XL automated epifluorescence microscope (Molecular Devices, San Jose, CA, USA) using a Plan Fluor ELWD 20 × /0.45 objective. The acquired images were analyzed as the combination of these two techniques by use of CellProfiler 4.2.0 open-source software (Boston, MA, USA; www.cellprofiler.org accessed on 15 July 2021) as well as manually by the use of FIJI software (Madison, WI, USA; www.fiji.sc accessed on 15 July 2021).

#### 2.3.3. MTT Cell Viability/Metabolic Activity Test

Viability/mitochondrial metabolic activity was measured by an assay using 3-(4,5-dimethylthiazol-2-yl)-2,5-diphenyltetrazolium bromide (MTT). The cells were incubated with 1 mg/mL of MTT dissolved in NSC medium at 37 °C for 1 h. Then, the NSC medium was aspirated and cells were treated with 99.9% DMSO for 10 min while being shaken. Absorbance was detected at 570 nm and 690 nm, as a reference, using a Synergy HTX spectrophotometer (BioTek Instruments, Inc., Winooski, VT, USA.).

#### 2.3.4. Flow Cytometry Analyses

For the flow cytometry analyses, the cells at the end of the treatment were harvested into the medium using Accutase, centrifuged at 200× *g* for 5 min at room temperature, washed with PBS, centrifuged again at 200× *g* for 5 min, and then fixed by the addition of 1 mL of ice-cold 70% ethanol by individual drops with gentle vortexing, and stored at 4 °C for at least 30 min. Before the flow cytometry analyses, fixed cells were centrifuged (200× *g* for 5 min), and the pellet was washed twice with FACS (Fluorescent Activated Cell Sorting) buffer (0.5 M EDTA, 2% FBS, 1 × PBS) and finally resuspended in 250 µL of FACS buffer with 50 µL of RNase A (0.1 mg/mL) for 30 min at 37 °C. The nuclei were stained by adding propidium iodide (Thermo Fisher Scientific, Waltham, MA, USA) to make a final concentration of 50 µg/mL. Samples were incubated in the dark for 30 min at room temperature and subjected to FACS analysis. Data were collected for a minimum of 6000 events per sample. Flow cytometry was performed by use of a BD FACS Canto II cytometer (Becton Dickinson, Franklin Lakes, NJ, USA) with excitation at 535 nm and emission at 617 nm. The analyses were performed with FlowJo 7.2.2 software (Tree Star, Ashland, OR, USA).

### 2.4. Western Blot Analysis

Samples of self-renewing NSC were harvested after 96 h of treatment with BR (12.5 µM) or LR (12.5 and 25 µM) and analyzed according to Fedorová et al. [21]. In brief, the NSC were washed once with PBS, lysed by 250 µL of lysis buffer (50 mM TRIS-HCl pH 6.8, 1% SDS, 10% glycerol), and stored at −20 °C. The protein concentration was measured by a DC Protein Assay Kit (BioRad, Prague, Czech Republic). Ten μg of total protein per well was separated using 8 to 10% SDS-polyacrylamide gel electrophoresis. The proteins were transferred to the PVDF (polyvinylidene fluoride) membrane (Merck, Millipore, MA, USA), blocked with 5% milk in Tris-buffered saline, and incubated overnight at 4 °C with the appropriate primary antibody. The membranes were incubated with secondary antibodies, and the proteins were visualized on AGFA film using Amersham ECL Prime Western blotting detection reagent (GE Healthcare Life Sciences, Marlborough, MA, USA). β-actin was used as a loading control. The list of antibodies is described in Table S1. The densitometry was performed using FIJI software, and the data were visualized using GraphPad Prism version 8.0.0 for Windows (GraphPad Software, San Diego, CA, USA; www.graphpad.com accessed on 15 July 2021).

### 2.5. DNA Damage Analysis (Comet Assay)

For these analyses, the NSC that were treated with H_2_O_2_ (3.7 ppm) for 10 min before harvesting were used as a positive control. The cells were detached from the surface by Accutase and harvested in the basic NSC medium without FGF2. After centrifugation at 200× *g* for 5 min, the supernatant was aspirated and cells were stored in the freezing medium (60% basic NSC medium, 30% FBS, 10% DMSO) in liquid nitrogen. To measure the genotoxicity, the cell suspension was centrifuged at 1000× *g* for 1 min, and the pellet was re-suspended in 0.5 mL of PBS. Fifty μL of the suspension was mixed with 150 μL of low melting point agarose (0.01 g/mL), and 80 μL of the resulting agarose-cell suspension was seeded on a precoated slide with 1% agarose. Cell lysis, gel electrophoresis, and staining were performed as previously described [22]. The slides were analyzed for the tail length and intensity using an ImageJ 1.51s (National Institutes of Health, Bethesda, MA, USA) connected to a fluorescence microscope (AX70 Provis, Olympus, Japan).

### 2.6. Immunofluorescence and Cell Imaging

For the cell imaging experiments, NSC cells were seeded at a density of either 12,000/cm^2^ or 25,000/cm^2^ (for differentiation to neurons) as previously described. After fixation with 4% paraformaldehyde, the cells were washed three times with PBS and permeabilized with buffer (0.2% Triton X100 with 1× PBS) for 15 min, and the primary antibodies were incubated overnight at 4 °C. Next, the slides were washed three times with PBS. Secondary antibodies and Hoechst were added with the permeabilization buffer and incubated for 1 h at room temperature (the antibodies’ dilutions are described in Table S2A,B). After incubation, the slides were washed three times with PBS, dried, and mounted onto microscopic slides with Mowiol 4-88 Reagent (Merck, Darmstadt, Germany). Cell imaging was performed with a Zeiss LSM 800 Laser scanning confocal microscope using an ORCA-Flash 4.0LT digital sCMOS monochromatic camera and Plan-Neofluar 20 × /0.50 AIR objective (Zeiss, Jena, Germany).

### 2.7. RNA Isolation and Quantitative Real-Time RT-PCR

Samples for analyses were harvested 12 and 17 days after the seeding of the NSC for terminal differentiation. Total RNA was isolated using RNA Blue reagent (Top-Bio, Prague, Czech Republic) according to the manufacturer’s instructions. The total amount of RNA was measured by a NanoDrop^®^ ND 1000 UV-Vis spectrophotometer (Thermo Fisher Scientific, Waltham, MA, USA). For quantitative real-time RT-PCR, cDNA was synthesized by a Transcriptor first-strand cDNA synthesis kit (Roche, Basel, Switzerland), and the analyses were performed in triplicates using SYBR Green I Master (Roche, Basel, Switzerland) according to the manufacturer’s instructions. The designed PCR primers by PrimerBank and OriGene are described in Table S3. DNA amplification was detected with 2^−^^ΔCt^ analysis using a LightCycler 480 II (Roche, Mannheim, Germany). The relative mRNA levels were normalized to GAPDH. The data were visualized using GraphPad Prism version 8.0.0 for Windows.

### 2.8. Statistical Analyses

All data are expressed as the mean ± standard deviation. Depending on their distribution, the data were assessed either by *t*-test or Mann–Whitney test using Prism 8.0.1. software (GraphPad, San Diego, CA, USA). Differences were considered statistically significant at a *p*-value ≤ 0.05.

## 3. Results

### 3.1. The Effect of BR and LR on the Viability of NSC

**Figure 1:**
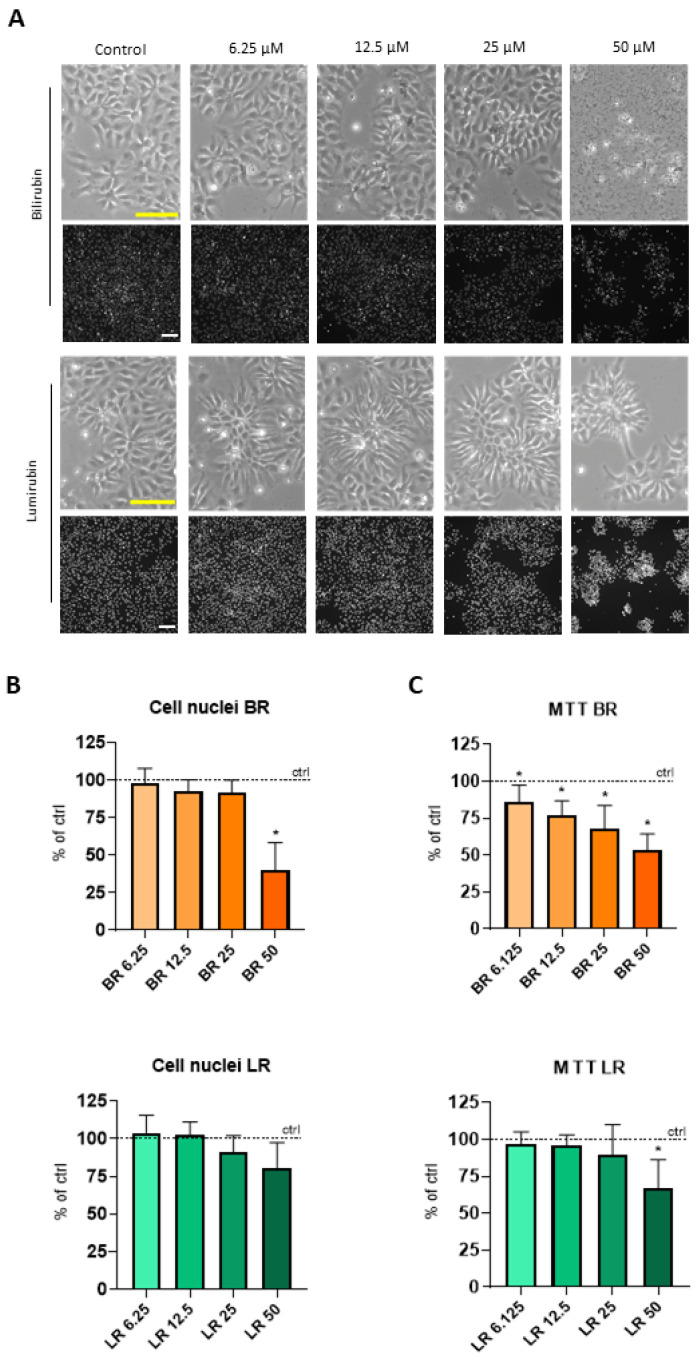
The effect of BR and LR on the viability of NSC. (**A**) Morphology of NSC exposed to BR and LR (brightfield images, scale bar = 250 µm) and Visualization of the cell nuclei (bottom black panels; scale bar = 150 µm, stained with Hoechst). (**B**) Cell nuclei quantification of NSC exposed to BR and LR. Stained by Hoechst and analyzed by CellProfiler and ImageJ. Controls expressed as 100%. *n* = 4. (**C**) Viability/metabolic activity of NSC exposed to BR and LR. Analyzed by MTT test. Controls expressed as 100%. *n* = 4, * *p* < 0.05, exposure time = 96 h.

In the initial phase of our studies on self-renewing NSC derived from hPSC, we intended to assess the possible cytotoxic effects of both BR and its major photoisomer LR. After 96 h of continuous exposure to BR or LR, a decreased number of the cells treated with higher concentrations of both pigments were clearly detectable; this effect was most pronounced with the 50 μM concentration of BR (Figure 1A—Brightfield panels).

To quantify this phenomenon, the number of cell nuclei was evaluated using image analysis and the MTT assay was also performed. A significantly decreased number of nuclei was observed in the cells after exposure to the 50 µM BR concentration (*p* < 0.05) (Figure 1A—upper black panel, and Figure 1B), which was also confirmed in the viability assay (MTT test). In this test, a gradual and significant decrease in the viability/metabolic activity of the cells exposed to BR was observed within the whole range of tested concentrations (6.25–50 µM) (Figure 1C). Compared to BR, the effect of LR on the viability/metabolic activity of the treated cells was much lower, and only visible at the highest concentration (50 µM) (Figure 1C), indicating the much higher toxicity of BR on NSC compared to LR, its photo-oxidation product.

### 3.2. The Effect of BR and LR on the Proliferation and Cell Cycle of NSC

Based on the morphology and data on the counting of nuclei shown in Figure 1, 12.5 µM concentrations of BR and LR were selected for all of the experiments that followed. For both BR and LR, the cells exposed to these concentrations retained a healthy morphology and the numbers of cell nuclei were comparable to those in the solvent controls. As the MTT assay is a measure of metabolic activity [14,23], we assumed that adverse effects detected after treatment with BR might have been affected by its impact on cellular metabolism rather than viability. In addition, it should be noted that the selected LR concentration is biologically relevant for hyperbilirubinemic neonates treated with PT, as proven in our previous clinical observation [8].

**Figure 2:**
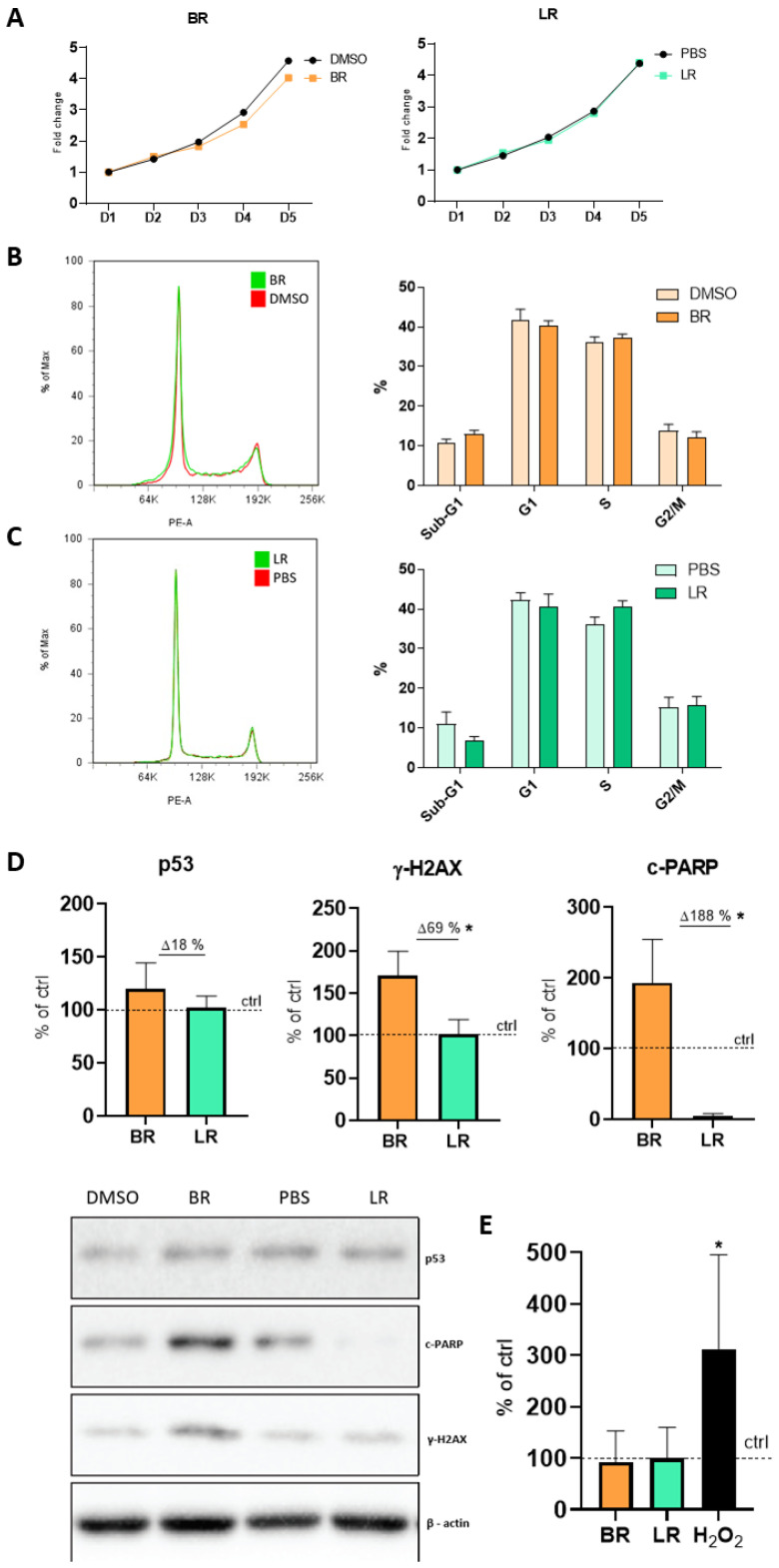
The effect of BR and LR exposure on behavior of NSC. (**A**) Growth curve of the NSC exposed BR. BR and LR concentration = 12.5 µM, 0.1% DMSO and 1% PBS used as respective controls. Exposure time = 96 h, *n* = 4. (**B**,**C**) Cell cycle analysis of NSC exposed to BR and LR. (**B**) 12.5 µM BR (BR 12.5) and 0.1% DMSO (BR control). *n* = 4; (**C**) 12.5 µM LR (LR 12.5) with 1% PBS (LR control). *n* = 4; charts represent the quantification of at least four independent measurements. BR and LR concentrations = 12.5 µM, 0.1% DMSO and 1% PBS used as respective controls. Exposure time = 96 h, *n* = 4. (**D**) Expression of p53, c-PARP, and γ-H2AX proteins in NSC exposed to BR and LR. BR and LR concentrations = 12.5 µM, 0.1% DMSO and 1% PBS used as the respective controls. β-actin was used as a loading control. Quantification of Western blots was performed using ImageJ. Exposure time = 96 h, *n* = 3, * *p* < 0.05. (**E**) The extent of DNA damage/single- and double-strand breaks of NSC exposed to BR and LR. Analyses performed by the Comet assay. BR and LR concentrations = 12.5 µM, 0.1% DMSO and 1% PBS used as respective controls, H_2_O_2_ was used as a positive control. Exposure time = 96 h, *n* = 3.

The growth curves constructed to analyze the effect of both pigments on the NSC proliferation rate did not demonstrate anything adverse during 96 h of exposure (Figure 2A); although a slight but a non-significant trend toward slower proliferation of BR-treated cells was detectable. Subsequent measurements of the cell cycle profile and distribution of cells in the cell cycle phases confirmed that NSC treated with BR or LR do not accumulate in any of the phases of the cell cycle (Figure 2B,C).

To further explore the possible effects of BR and LR on the NSC, the protein expressions of the following apoptotic or DNA damage-related markers were analyzed in NSC exposed to both pigments for 96 h: tumor suppressor protein p53, cleaved nuclear poly (ADP-ribose) polymerase (c-PARP), and the phosphorylated form of H2A histone family member X (γ-H2AX). Although no significant changes in the expression of p53 were detectable in the cells, a significantly increased c-PARP (*p* < 0.05) and γ-H2AX (*p* < 0.05) protein expression was observed in NSC exposed to BR. Conversely, a significant and completely opposite trend of the expression of c-PARP in NSC exposed to LR was detected (*p* < 0.05, Figure 2D). Lastly, in this set of experiments the extent of DNA damage/single- and double-strand breaks in the cells exposed to BR and LR was quantified by Comet assay. Nevertheless, no significant changes in these parameters of NSC exposed to both pigments were observed (Figure 2E). Overall, our results demonstrated that the proliferation and cell cycle of NSC were not significantly altered after exposure to BR or LR. However, substantial changes were detectable on a molecular level where BR exposure induced apoptotic (c-PARP) and DNA damage (γ-H2AX) markers, while LR-exposure in clinically relevant concentrations exerted protective effects against these changes.

### 3.3. LR Induces Morphological Changes and Rosette Formation in Self-Renewing NSC

While testing the toxicity of various concentrations of LR on NSC (as shown in Figure 1A), we noticed that with increasing concentrations of LR, the NSC significantly changed their undifferentiated arrangement and acquired a different phenotype.

**Figure 3:**
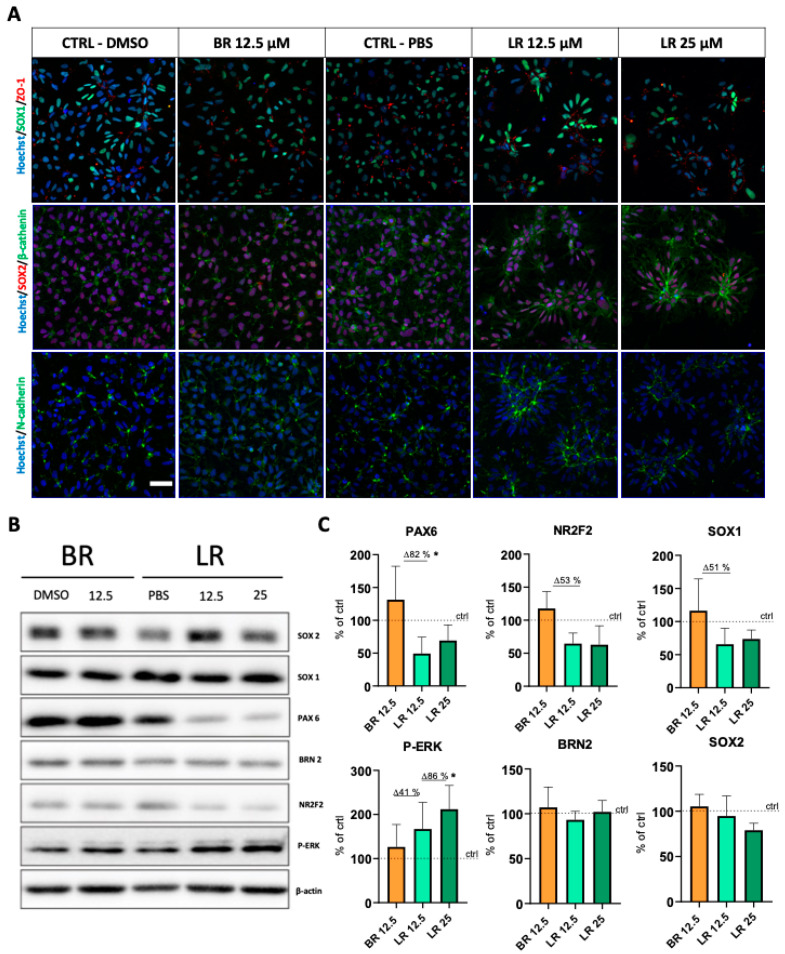
The effect of BR and LR on the NSC differentiation markers. (**A**) Immunostaining of apically localized cell polarity proteins of NSC exposed to BR and LR. Staining and visualization was performed after 96 h exposure of the NSC to 12.5 µM BR (BR 12.5) with 0.1% DMSO (BR control), 12.5/25 µM LR (LR 12.5/25) with 1% PBS (LR control). Cells were labeled by three combinations (1) SOX1 (green) and ZO-1 (red), (2) SOX2 (red) and β-catenin (green), and (3) N-cadherin (green). Nuclei were counterstained with Hoechst (blue) and visualized with a Zeiss LSM 800. Scale bar = 50 µm. (**B**) Western Blot analysis of NSC-specific transcription factors. Data were normalized first to β-actin (used here as a loading control), and then visualized as percentage of respective control. (**C**) Quantification of protein expressions of NSC-specific transcription factors. Quantification of Western blot signals performed using ImageJ. *n* = 3. * *p* < 0.05.

In order to explore this phenomenon in greater detail, immunofluorescent staining of those markers typical for the rosette structures was performed to confirm this observation. Specifically, we detected substantial changes in the N-cadherin, zonula occludens-1 (ZO-1), and β-catenin distribution within the NSC, all markers of the formation of neural rosettes (Figure 3A) [24,25]. As visualized by immunostaining, the cells retained the expression of SOX1 and SOX2, the crucial transcription factors (Figure 3A) [25,26]. These changes were clearly visible in LR at a concentration of 12.5 µM and were even more pronounced when using 2-fold higher concentration. Our data indicated that NSC treated with LR showed a significantly polarized phenotype, typical for immature neural rosettes, being even more apparent with higher concentrations of LR. Importantly, this phenomenon was unique to LR; it was not detected in NSC exposed to BR.

Based on these results, Western blot analysis of NSC-specific transcription factors was performed. These included those expressed upon differentiation of hPSC towards neuroectoderm, such as PAX6, SOX1, SOX2, BRN2, and NR2F2 [21]. Interestingly, compared to BR treatment, expressions of NSC-specific markers upon exposure to LR showed no change (BRN2, SOX2), a minor decrease (SOX1, NR2F2), or a significant drop (PAX6; *p* < 0.05) (Figure 3B,C). In addition, we also analyzed ERK activation (ERK/P-ERK), a signaling pathway important for neural cell differentiation from NSC [27]. Interestingly, a significant and dose-dependent upregulation of P-ERK expression was clearly present upon exposure of the NSC to LR.

The data strongly suggested that LR-treated self-renewing NSC acquire a significantly different morphology reminiscent of immature rosettes, with apically localized cell polarity proteins; a phenomenon accompanied by changes in various transcription factors and activation of an ERK signaling pathway.

### 3.4. The Effect of BR and LR Exposure on Terminal Differentiation of NSC

**Figure 4:**
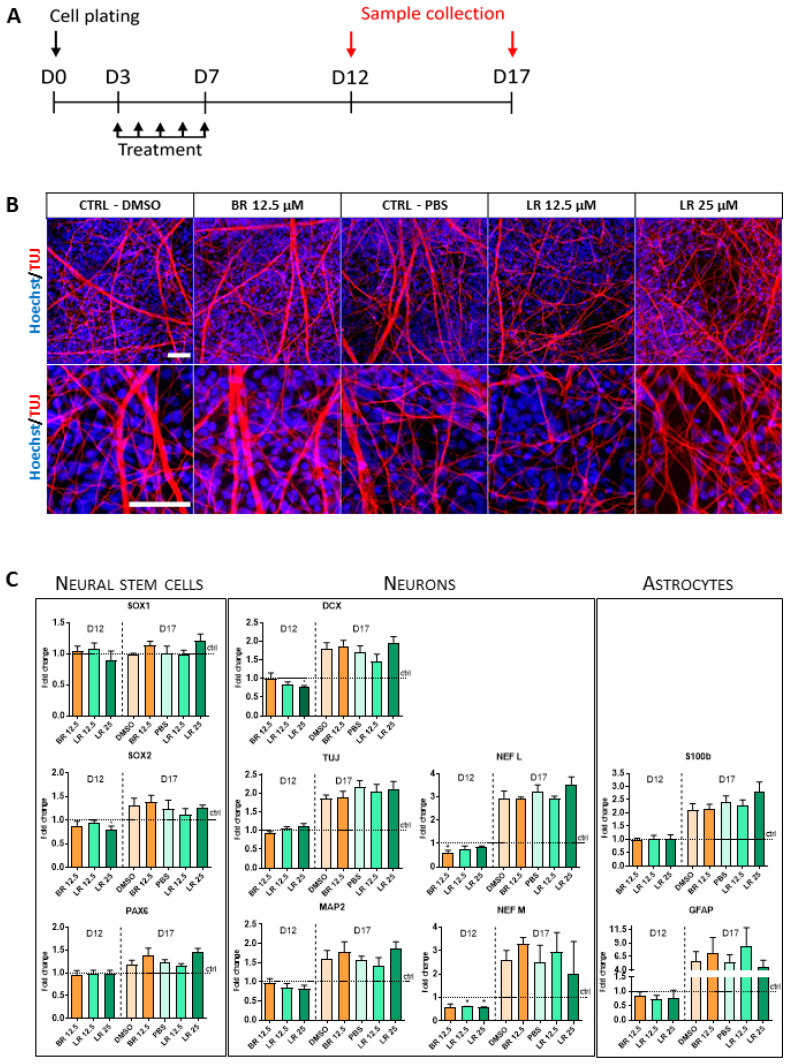
The effect of BR and LR on terminal differentiation of NSC. (**A**) Experimental layout. (**B**) Tubulin-positive neuronal filament formation upon exposure to BR and LR. Visualization was performed after 17 days of terminal differentiation, when the NSC were treated with 12.5 µM BR (BR 12.5) and 0.1% DMSO as a control, and 12.5 µM LR (LR 12.5) and 25 µM LR (LR 25) with 1% PBS as a control. Cells were stained by Hoechst (blue) and TUJ (red) and visualized with a Zeiss LSM 800. Scale bars = 50 µm. (**C**) The effect of BR and LR exposure on the expression of selected markers specific for NSC, neurons, and astrocytes. Samples were harvested on Day 12 and 17 of terminal differentiation. * *p* < 0.05. Two different time points were chosen to evaluate possible changes in the onset/timing of the differentiation process. Genes were detected using a LightCycler 480 II. Relative mRNA levels were normalized to GAPDH with 2^−^^ΔCt^ analysis. *n* = 3.

Lastly, in order to determine if BR or LR affected the ability of the NSC to terminally differentiate into neurons, the NSC were exposed to both pigments and the extent of their terminal differentiation was subsequently assessed. NSC were seeded in a standard conditioned growth medium on Day 0, and the treatment was started three days after plating (for details of the experimental design see Figure 4A).

First, the general morphology of the neurons was evaluated using immunofluorescence staining at the end of our differentiation protocol. As shown in Figure 4B, differentiating NSC formed a homogeneously distributed network of tubulin (TUJ)-positive neuronal filaments. However, no significant morphological differences between BR- or LR-treated cells (and their respective solvent controls) were detectable in these experiments. The gene expression of selected differentiation-associated markers was then analyzed. The results showed that the NSC had gradually increased the expression of selected neuronal markers (such as DCX, TUJ, MAP2, NFL, and NFM) as well as glial markers (S100β and GFAP), while there were no changes in the expression of the neural stem cell markers (SOX2, SOX1, and PAX6). However, no significant changes in the expression of any of these analyzed markers were observed after treatment with BR or LR. This is certainly a notable observation, since just a short-term exposure led to significant changes in these markers (Figure 3C), suggesting these pigments played a role in the earlier phases of differentiation of the NSC, an influence which, however, was later lost. Altogether, our data suggest that despite visible changes in the morphology of NSC, no major changes can be detected at the level of the terminal differentiation of NSC towards neuronal and glial cell types.

## 4. Discussion

Severe neonatal hyperbilirubinemia represents a serious health threat for newborn infants with global health and socioeconomic burdens [2,28]. Although PT is considered the gold standard treatment for severely jaundiced neonates, there is a surprising lack of scientific data on the biological effects of bilirubin photo-oxidation products generated during PT. Importantly, PT does not seem to be entirely safe, particularly in ELBW preterm neonates [10,11].

As was demonstrated in our recent study, a short-term 4-h exposure to BR and its photoisomer LR, as well as monopyrrolic BOX-A and BOX-B of human neuroblastoma (SH-SY5Y), glioblastoma (U-87), and microglial cells (HMC3) (representing major brain cerebral cell populations), did not affect cell viability at all, even at the biologically relevant concentrations (25 µM) [13]. However, prolongation of exposure of the neuronal cells up to 24–48 h revealed clear BR toxicity, while LR did not have this toxic effect [14]. One of the reasons for these observations lies in the profound inhibitory effects of BR on mitochondrial respiration and the tricarboxylic acid cycle metabolism [14]. BR neurotoxicity has also been confirmed in more complex studies, such as those on organotypic brain slices derived from hyperbilirubinemic Gunn rat pups [29], plus other experimental studies (for review of the mechanisms see [5]), as well as in numerous clinical observations [2,28]. These findings are consistent with the early studies by Silberberg et al., who did not detect any toxic effects of photo-irradiated bilirubin on myelinating cerebellum cultures–in contrast to bilirubin [30]. However, LR (and also the other BR photo-oxidation products) do not appear to be harmless. In another of our recent studies, the potential neuroinflammatory effects of LR as evidenced by overexpression of proinflammatory cytokines in brain cells were clearly documented [13]. It is important to note that numerous studies confirmed proinflammatory cytokines exerting harmful effects on neurogenesis [31]; these results are in agreement with the clinical data on hyperbilirubinemic newborn infants treated with PT, which demonstrate an increase in systemic concentrations of circulating proinflammatory cytokines [32].

Based on these findings, and since NSC have never been studied for their susceptibility to the potentially harmful effects of BR, we focused on the possible cytotoxic effects of BR and LR on these cells. We observed a significantly decreased viability and metabolic activity in those cells treated with gradually increasing concentrations of BR, while LR only had a significant effect only at the highest concentration. 

As discussed above, BR in high concentrations clearly exerts cytotoxicity, in particular on cells of the central nervous system (CNS). However, the pathogenesis of the whole process is not fully understood, with significant differences in susceptibility to BR toxicity across various brain regions and cell populations [29,33]. Previous studies have also shown that exposure to increasing concentrations of unconjugated BR are cytotoxic to rat oligodendrocytes and increase its apoptosis in vitro [34]. Several additional studies have shown the cytotoxic and pro-apoptotic effects of BR on astrocytes’ neuronal cultures [35,36,37,38,39]. Although under our study conditions no significant changes in DNA damage were observed using the Comet assay, and the flow cytometry analysis demonstrated only a negligible modulation of the cell cycle of treated NSC exposed to BR, an increase in c-PARP (apoptotic), γ-H2AX (DNA damage), and p53 (apoptosis activator) protein expression was clearly demonstrable, consistent with previous reports mapping BR-induced apoptosis and DNA fragmentation in rat brain neurons [40]. Importantly, these changes in NSC were prevented by the biologically relevant concentration of LR (12.5 µM), suggesting the neuroprotective effects of LR. Hence, changes at the molecular level may not be apparent when using non-specific methods, such as the Comet assay (used in our study), where the negative result could be explained by its decreased sensitivity during the analysis of small pro-apoptotic changes [41]. On the other hand, the data are in contrast with observations on DNA damage in peripheral blood lymphocytes [42,43,44,45] and increased serum apoptotic markers [46] in phototherapy-treated newborn infants suggesting cell-specific responsiveness to BR and LR.

Interestingly, while performing these studies on the cytotoxicity of BR and LR, we noticed that LR induced major morphological changes in our self-renewing NSC, while no such changes were noticed after exposure to BR. This observation may be of clinical importance, since cellular polarity plays a significant role during the development of the CNS [47]. During the onset of neural differentiation in vivo, the neuroepithelium forming the neural tube represents the first polarized single cell layer with a central lumen and cells displaying apicobasal polarity [48]. Under in vitro conditions, this phenomenon is mimicked by the formation of neural rosettes—radially organized neuroepithelial cells differentiated from hPSC [21,48,49,50]. Such polarity also ensures a different distribution of junction proteins (such as N-cadherin, ZO-1, and β-catenin) as well as of signaling molecules including Notch, Sonic Hedgehog, or FGF2/ERK [24,49,50]. Importantly, the rosette structures also represent the niche from which NSC are isolated. Surprisingly, our study demonstrated for the first time that LR induces NSC to repolarize, and that this induction is dose-dependent. Additionally, these repolarized NSC cultures, possibly as a positive feedback mechanism, expressed higher amounts of phosphorylated ERK, important for the process of neurogenesis [27], as well as showing an altered expression of NSC-specific markers including PAX6, NR2F2, and SOX1. Thus, our data suggested that LR has the potential to affect the polarity and identity of NSC during early human neural development. These results may be of clinical relevance, since aggressive PT is used on preterm ELBW neonates, often accompanied by serious adverse effects [10,11]. It is also well known that the processes of neurogenesis and neurodevelopment are impaired in these neonates [51], which may even be exacerbated by BR photo-oxidation products generated during PT, based on our current data.

Finally, we also assessed the capacity of BR or LR to affect the terminal differentiation of NSC. Previously, it has been shown that moderate to severe hyperbilirubinemia could induce neurological dysfunction and potentially impair brain myelination with long-term sequelae, particularly in preterm infants [6]. However, studies addressing the possible effects of LR and/or other BR photo-oxidation products so far have not been reported. Here, we found that despite significant changes in the expression of pro-apoptotic markers in BR-exposed NSC, or altered cell polarity and morphology in LR-exposed NSC, no major changes were detected in differentiating neurons or in their gene expressions.

## 5. Conclusions

Our study has assessed the possible impact of BR as well as its major photoisomer LR on human neurodevelopment using an in vitro model of hPSC-derived NSC. Our data demonstrate that neither of these compounds significantly affected the extent of terminal differentiation of neurons and astrocytes. However, compared to LR, BR exerted a higher cytotoxicity on self-renewing NSC, and this effect was dose-dependent and accompanied by mildly elevated pro-apoptotic markers. On the other hand, LR had a dose-dependent effect on the morphology of self-renewing NSC, inducing them to form highly polarized structures with lower expressions of some NSC-specific markers, but with activation of ERK signaling. Considering the tightly orchestrated processes during the formation of the CNS, this effect of changed cell polarity and identity could potentially have a detrimental effect on the developing brain. Although complex mechanisms behind these phenomena remain to be addressed, both BR and LR clearly affect early human neurodevelopment in vitro.

## Data Availability

All research data are available on request from the Corresponding Author. We do not have webpages created for such purpose, but as clearly stated the data are available on request and can be sent to anyone interested in our research.

## Supplementary Materials

The following are available online at https://www.mdpi.com/article/10.3390/antiox10101532/s1, Table S1: List of antibodies used for Western blot analyses, Table S2: List of primary and secondary antibodies used for the studies, Table S3: List of genes used for gene expression analyses.
