# Supplementary material for: The Effects of Bilirubin and Lumirubin on the Differentiation of Human Pluripotent Cell-Derived Neural Stem Cells
Source: Antioxidants (Basel). 2021 Sep 27;10(10):1532. doi: 10.3390/antiox10101532 (PMC8532948; doi:10.3390/antiox10101532)
Supplement: Supplementary file 1 [file antioxidants-10-01532-s001.zip › antioxidants-1377875-supplementary.pdf]

Table S1. List of antibodies used for Western blot analyses.

Antibody	Manufacturer
p53 (DO-1)	Kindly provided by Bořivoj Vojtěšek
Cleaved Parp (D64E10)	Cell Signaling (MA, USA)
phospho-gH2AX (20E3)	Cell Signaling
P-p44/42 MAPK (p-ERK)	Cell Signaling
Pax6 (D3A9V)	Cell Signaling
Sox1	Cell Signaling
Sox2 (L1D6A2)	Cell Signaling
NR2F2 (Coup-TFII) (D16C4)	Cell Signaling
Brn2/Pou3f2 (D2C1L)	Cell Signaling

Table S2. List of primary and secondary antibodies used for the studies.

Primary antibody	Dilution	Secondary Antibody	Dilution
SOX1	1:500	AlexaFluor 488	1:500
ZO1	1:250	AlexaFluor 568	1:500
SOX2	1:500	AlexaFluor 568	1:500
ß-cathenin	1:500	AlexaFluor 488	1:500
N-cadherin	1:500	AlexaFluor 488	1:500
DAPI	1:1,500	-	-
ß3-tubulin	1:500	AlexaFluor 568	1:500
DAPI	1:1500	-	-

Table S3. List of genes used for gene expression analyses.

Gene	Protein	Forward Primer	Reverse Primer
GADPH	Glyceraldehyde-3-phosphate dehydrogenase	agccacatcgctcagacac	gcccaatacgaccaaatcc
NEFL	Neurofilament light polypeptide	cgacagcttgatggacgaaat	gatctgcgcgtactggatctg
NEFM	Neurofilament medium polypeptide	gaaatcgctgcgtacagaaaac	taatggctgtcagggcctctt
NEFH	Neurofilament heavy polypeptide	gcagtccgaggagtggttc	cgcatagcgtctgtgttca
SOX1	Transcription factor SOX-1	tcccccgcgtgaactg	caaggcattttgcgttcaca
SOX2	Transcription factor SOX-2	tacagcatgtcctactcgcag	gaggaagaggtaaccacaggg
PAX6	Paired box protein Pax-6	ctgaggaatcagagaagacaggc	atggagccagatgtgaaggagg
DCX	Neuronal migration protein doublecortin	tatgcgccgaagcaagtctcca	catccaaggacagaggcaggta
TUBB3	Tubulin beta-3 chain	tcagcgtctactacaacgaggc	gcctgaagagatgtccaaaggc
MAP2	Microtubule-associated protein 2	ttggtgccgagtgagaaga	gtctggcagtggttggttaa
GFAP	Glial fibrillary acidic protein	ccgacagcaggtccatgt	gttgctggacgccattg
